# Lochmolins A–G, New Sesquiterpenoids from the Soft Coral *Sinularia lochmodes*

**DOI:** 10.3390/md10071572

**Published:** 2012-07-20

**Authors:** Yen-Ju Tseng, Kuo-Ping Shen, Hui-Li Lin, Chiung-Yao Huang, Chang-Feng Dai, Jyh-Horng Sheu

**Affiliations:** 1 Department of Marine Biotechnology and Resources, National Sun Yat-sen University, Kaohsiung 804, Taiwan; Email: pit0424@yahoo.com.tw (Y.-J.T.); betty8575@yahoo.com.tw (C.-Y.H.); 2 Department of Nursing, Meiho University, Pingtung 912, Taiwan; Email: x00002148@meiho.edu.tw; 3 Department of Food and Nutrition, Meiho University, Pingtung 912, Taiwan; Email: x00002165@meiho.edu.tw; 4 Institute of Oceanography, National Taiwan University, Taipei 112, Taiwan; Email: corallab@ntu.edu.tw; 5 Division of Marine Biotechnology, Asia-Pacific Ocean Research Center, National Sun Yat-sen University, Kaohsiung 804, Taiwan

**Keywords:** soft coral, *Sinularia lochmodes*, sesquiterpenes, aromadendrane, germacrane

## Abstract

Seven new sesquiterpenoids, lochmolins A–G (**1**–**7**), were isolated from a Taiwanese soft coral *Sinularia lochmodes*. The structures of these metabolites were elucidated by extensive spectroscopic study. Compounds **1**–**4** were found to inhibit the accumulation of the LPS-induced pro-inflammatory COX-2 protein in RAW264.7 macrophage cells.

## 1. Introduction

**Chart 1:**
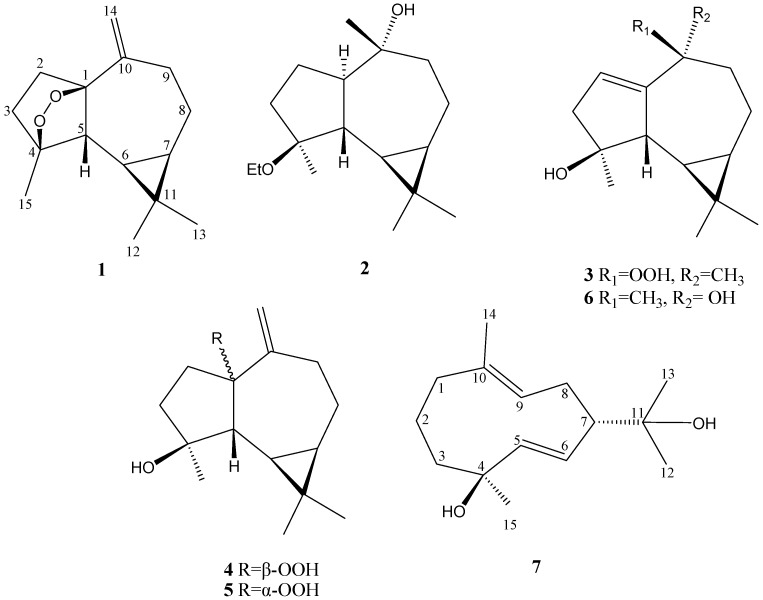
Structures of metabolites **1**–**7**.

Soft corals of the genue *Sinularia* have been discovered to be a rich source of terpenes [1]. Previously, we discovered a 9,11-secosterol [2] and diterpenes-related compounds [3,4] from the soft coral *Sinularia lochmodes* collected off the coast of southern Taiwan. Our current chemical investigation of the soft coral *S. lochmodes*, collected from the northeastern coast of Taiwan, has led to the isolation of six new aromadendrane-type [5,6,7] sesquiterpenoids lochmolins A–F (**1**–**6**), and a new germacrane [8] sesquiterpenoid lochmolin G (**7**) (Chart 1). The relative structures of the new metabolites were established by extensive spectroscopic analysis. The ability of **1**–**7** to inhibit up-regulation of the pro-inflammatory iNOS (inducible nitric oxide synthase) and COX-2 (cyclooxygenase-2) proteins in LPS (lipopolysaccharide)-stimulated RAW264.7 macrophage cells was also evaluated.

## 2. Results and Discussion

**Figure 1:**
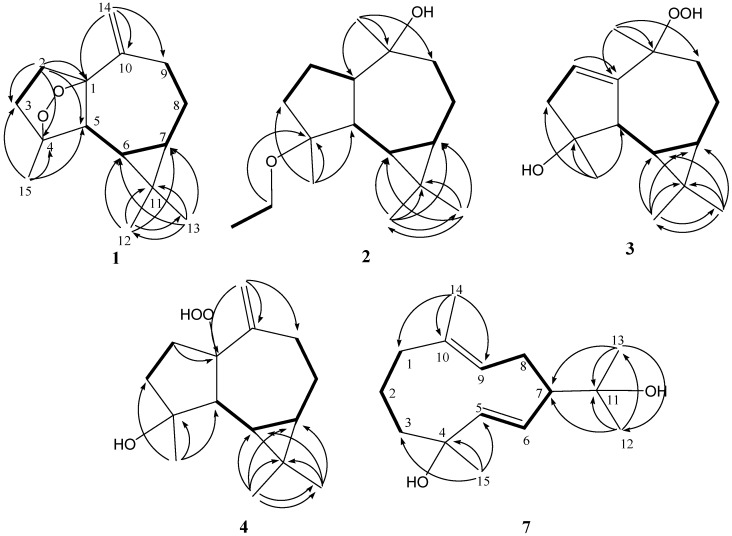
Selected ^1^H-^1^H COSY (▬) and HMBC (→) correlations of **1**–**4** and **7**.

Lochmolin A (**1**) was isolated as a colorless oil. Its molecular formula, C_15_H_22_O_2_, was established by HREIMS (*m/z* 234.1620, [M]^+^), implying five degrees of unsaturation. The ^13^C NMR spectral data of **1** (Table 1), showed the presence of 15 carbon atoms, including three methyls (δ_C_ 28.5, 24.8, and 16.2) and two quaternary sp^3^oxycarbons (δ_C_ 89.3 and 83.1), as assigned by the DEPT spectrum, suggesting the oxygenated sesquiterpenoid nature of **1**. The NMR signals (Table 1 and Table 2) observed at δ_C_ 112.7 (CH_2_) and 151.5 (C), δ_H_ 5.02 and 4.89 (each 1H, s) showed the presence of one 1,1-disubstituted double bond. Thus, the tetracyclic structure of **1** was revealed. In the ^1^H-^1^H COSY spectrum it was possible to identify two different structural units, which were assembled with the assistance of an HMBC experiment. Key HMBC correlations of H_2_-2 to C-1, C-3, C-4 and C-5; H_3_-12 to C-6, C-7, C-11 and C-13; H_3_-13 to C-6, C-7, C-11 and C-12; H_2_-14 to C-1, C-9 and C-10; H_3_-15 to C-3, C-4 and C-5 permitted the establishment of the aromadendrane-type skeleton of **1** (Figure 1). Furthermore, the two additional oxygen atoms could be used to form an endoperoxide bridge in the cyclopentane moiety of the molecule from the downfield chemical shifts of the sp^3^ carbons C-1 (δ 89.3, C) and C-4 (δ 83.1, C). The presence of a cyclopropane was further confirmed by the upfield chemical shifts of H-6 (δ 0.08) and H-7 (δ 0.54). 

**Table 1:** ^13^C NMR spectroscopic data for compounds **1**–**7**.

	1	2	3	4	5	6	7
1	89.3	47.3	150.1	101.1	96.8	155.2	41.1
2	36.0	23.4	125.2	29.6	29.1	117.3	23.4
3	38.9	37.8	45.3	39.0	39.6	45.1	40.8
4	83.1	79.3	82.2	81.7	81.0	82.2	73.0
5	56.2	49.9	52.6	55.2	57.3	53.8	143.1
6	27.4	25.1	27.4	26.6	21.4	27.2	128.4
7	23.0	26.2	27.0	24.2	27.6	27.4	57.9
8	20.9	20.1	18.8	20.6	25.2	20.2	24.7
9	31.4	40.3	38.3	33.1	34.1	43.4	129.5
10	151.5	80.4	83.0	147.1	150.7	73.9	131.9
11	18.4	19.0	20.3	18.5	19.9	19.0	71.9
12	28.5	28.9	28.5	28.4	28.6	28.3	26.8
13	16.2	16.4	16.0	16.0	15.7	16.0	26.9
14	112.7	25.7	23.0	116.2	113.3	27.3	16.6
15	24.8	18.8	22.9	24.3	27.6	22.4	23.4
OEt		55.2					
		16.3					

**Table 2:** ^1^H NMR spectral data for compounds **1**–**4**.

	1	2	3	4
1		1.05 m		
2	2.01 m2.45 m	1.70 m1.80 m	5.66 d (3.0)	2.19 m2.46 m
3	1.95 m2.05 m	1.62 m1.72 m	2.27 dd (17.0, 3.0)2.61 d (17.0)	1.91 m1.97 m
5	1.77 d (12.0) *^a^*	2.38 dd (11.0, 5.5)	2.31 d (11.0)	1.68 d (11.5)
6	0.08 dd (12.0, 10.0)	0.64 m	0.27 dd (11.0, 9.5)	0.19 dd (11.5, 9.0)
7	0.54 dd (17.0, 10.0)	0.67 m	0.53 m	0.58 m
8	1.48 m1.87 m	0.89 m1.86 m	1.46 m1.65 m	1.40 m1.80 m
9	2.30 m2.65 m	1.48 m1.68 m	1.64 m1.92 dd (14.5, 5.5)	2.35 m2.63 m
12	1.00 s	1.05 s	1.02 s	1.02 s
13	1.06 s	0.97 s	1.12 s	1.04 s
14	4.89 s5.02 s	1.24 s	1.49 s	5.08 s5.14 s
15	1.30 s	1.05 s	1.40 s	1.27 s
16		3.40 m3.44 m		
17		1.12 t (7.0)		
10-OOH			7.64 s	

*^a^* *J* values (Hz) in parentheses.

**Figure 2:**
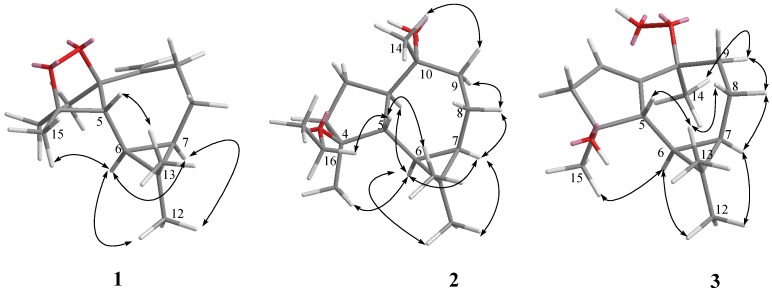
Key NOESY correlations for **1**–**3**.

The relative configurations of five chiral centers at C-1, C-4, C-5, C-6, and C-7 in **1** were elucidated by NOE analysis (Figure 2). It was found that H-6 showed NOE correlations with H-7, H_3_-12, and H_3_-15; H-7 (δ 0.54) showed NOE correlations with H_3_-12; and H-5 (δ 1.77) showed NOE correlations with H_3_-13. Thus H-6, H-7, and H_3_-15 were assumed to be positioned on the α face, and H-5 was assumed to be positioned on the β face. On the basis of these results, lochmolin A (**1**) was found to possess the (1R*,4S*,5R*,6R*,7R*) configuration.

Lochmolin B (**2**) was obtained as a colorless oil. HRESIMS showed the molecular formula C_17_H_30_O_2_, requiring three degrees of unsaturation. The IR spectrum suggested the presence of hydroxy group (3437 cm^−1^). The 3H triplet appearing at δ 1.12 (*J* = 7.0 Hz) in the ^1^H NMR spectrum and the methylene carbon signal at δ 55.2 in the ^13^C NMR spectrum were ascribable to an ethoxy group. Comparison of the NMR data (Table 1) of **2** with those of **1** also showed the aromadendrane skeleton of **2**. In the 2D NMR spectra, including ^1^H-^1^H COSY and HMBC (Figure 1), three segregate consecutive proton spin systems, H-1 to H_2_-3, H-5 to H_2_-9, and CH_2_ to CH_3_ of an ethoxy group, were found in the ^1^H-^1^H COSY spectrum. The detailed analysis of HMBC correlations further established the planar structure of **2**. The relative structure of **2** was elucidated by the analysis of NOE correlations, as shown in Figure 2. It was found that H-6 (δ 0.64, m) showed NOE interactions with H-1 (δ 1.05, m), H-7 (δ 0.67, m), H_3_-12 (δ 1.05, s) and H_3_-15 (δ 1.05, s), and H-7 (δ 0.67, m) showed NOE correlations with H-8α (δ 1.86, m) and H_3_-12 (δ 1.05, s); therefore, assuming the α-orientation of H-1, all of H-6, H-7, H_3_-12 and H_3_-15 should also be positioned on the α face. One of the methylene protons at C-9 (δ 1.68, m) exhibited NOE correlations with H-8α (δ 1.86, m) and was characterized as H-9α, while the other (δ 1.48, m) was assigned as H-9β. NOE correlations observed between H-9β and H_3_-14, and H-5 with both H_3_-13 (δ 0.97, s) and protons of OCH_2_, reflected the β-orientation of H-5 and H_3_-14. On the basis of the above findings (Figure 2), the relative structure of lochmolin B (**2**) was determined.

The HRESIMS spectrum of lochmolin C (**3**) exhibited a molecular ion peak at *m/z* 275.1622 ([M + Na]^+^), consistent with the molecular formula C_15_H_24_O_3_ and implying four degrees of unsaturation. The IR absorption of **3** also revealed the presence of hydroxy group (3437 cm^−1^). Comparison of the NMR data (Table 1 and Table 2) of **3** with those of **2** showed the appearance of an additional trisubstituted double bond in **3**. The NMR chemical shifts for C-4 and C-10 of **3** (δ 82.2 and δ 83.0, respectively), were found to be shifted downfield in comparison with the analogous data of **2** (δ 79.3 and δ 80.4), suggesting that the 4-OEt and 10-OH of **2** might be replaced by the 4-OH and 10-OOH (δ 7.64, s) in **3**. This could be confirmed from the carbon shifts of both hydroxylated quaternary carbons C-4 (δ 82.2) and C-10 (δ 73.9) of **6** (latter discussed) which showed the identical chemical shift of C-4 of compound **3**. By analysis of 2D NMR spectra (HMQC, ^1^H-^1^H COSY, and HMBC), compound **3** was shown to possess the same molecular framework as that of **2**. Investigation of the NOESY spectrum of **3** (Figure 2) revealed the NOE interactions of H-7 (δ 0.53, m) with H-8α (δ 1.65, m), H-8α with H-9α (δ 1.92, dd, *J* = 14.5, 5.5 Hz), and H-9α with H_3_-14 (δ 1.49, s), suggesting the α-orientation of H_3_-14. Further analysis of other NOE interactions revealed that **3** possessed the same relative configurations at C-4, C-5, C-6, and C-7 as those of **2**.

**Figure 3:**
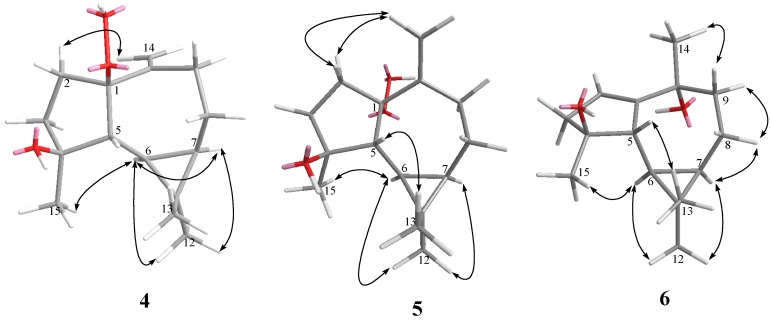
Key NOESY correlations for **4**–**6**.

Lochmolin D (**4**) was also isolated as a colorless oil with a molecular formula of C_15_H_24_O_3_. The ESIMS and NMR spectroscopic data of **4** (Table 1) showed the presence of a hydroxy and hydroperoxy moiety [δ 81.7 (C), and 101.1 (C)]. Comparison of the NMR data of **4** with those **1** revealed that the two differences between both compounds were the replacement of the endoperoxide bridge moiety at C-1 and C-4 in **1** by the hydroxy at C-4 and the hydroperoxy at C-1 in **4**. The relative structure of **4** was elucidated by the analysis of NOE correlations, as shown in Figure 3. It was found that H-6 (δ 0.19, dd, *J* = 11.5, 9.0 Hz) showed NOE interactions with H-7 (δ 0.58, m), H_3_-12 (δ 1.02, s), and H_3_-15 (δ 1.27, s), but not with H-5 (δ 1.68, d, *J* = 9.0 Hz); therefore, assuming an α-orientation of H-6, H-7 and H_3_-15 should also be positioned on the α face, and H-5 should be placed on the β face. One of the sp^3^methylene proton at C-2 (δ 2.19, m) exhibited NOE correlations with one of the sp^2^methylene proton at C-14 (δ 5.08, s), suggesting the β-orientation of 1-OOH by inspecting the molecular model of **4**. If the 1-OOH was placed on the α face as in the case of **5** (latter discussed), both protons at C-2 were found to exhibit NOE correlations with one of the sp^2^ proton at C-14 by molecular modeling study. On the basis of the above findings, the relative structure of **4** was determined.

HRESIMS and NMR spectroscopic data (Table 1 and Table 3) revealed that lochmolin E (**5**) has the same molecular formula, C_15_H_24_O_3_, as that of **4**. By analysis of 2D NMR spectra, including ^1^H-^1^H COSY, HMQC, and HMBC, compound **5** was shown to possess the same molecular framework as that of **4**. Comparison of the NMR data of **5** with those of **4** revealed that the only difference between the compounds was the replacement of the β-hydroperoxy group at C-1 in **4** by the α-hydroperoxy group in **5**. From the NOESY spectrum, it was found both protons of H_2_-2 (each 1H, δ 2.19 and 2.24, m) showed NOE interactions with one of the sp^2^methylene proton at C-14 (δ 4.97, s), suggesting the α-orientation of 1-OOH by investigation of the molecular model (Figure 3). Further analysis of other NOE interactions revealed that **5** possesses the same relative configurations at C-4, C-5, C-6, and C-7, as those of **4**. Therefore, **5**was found to be the C-1 epimer of **4**.

Lochmolin F (**6**) was obtained as a colorless oil and exhibited an ion peak at *m/z* 236.1774 ([M]^+^) by HREIMS, appropriate for the molecular formula C_15_H_24_O_2_. Comparison of the NMR data of **6** with those of **3** revealed that the only difference between both compounds was the replacement of a hydroperoxy group at C-10 in **3** by the hydroxy moiety in **6**. This was evidenced from the upfield chemical shifts induced by a hydroxy group at C-10 (δc 73.9) and H_3_-14 (δ_H_ 1.32) in **6** relative to those of **3**. The relative configuration of **6** was determined by analysis of key NOE correlations (Figure 3).

**Figure 4:**
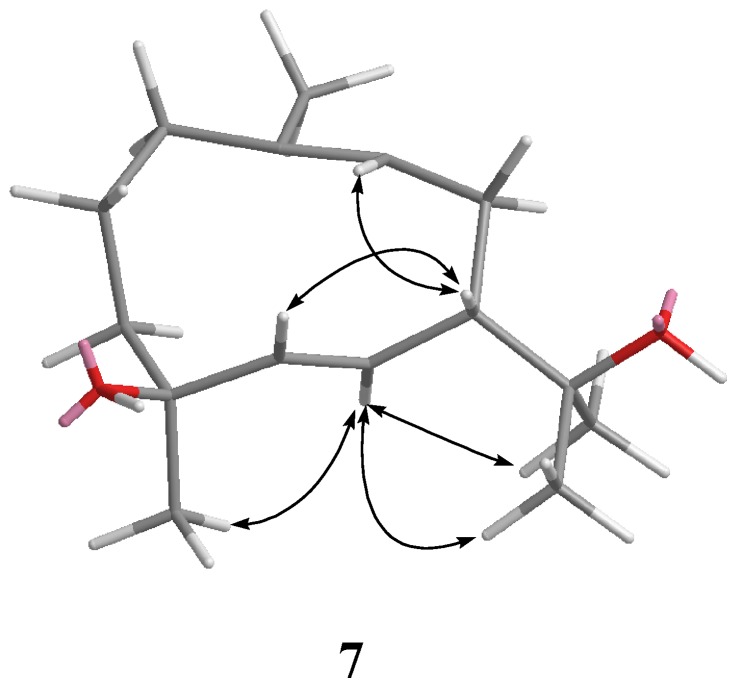
Key NOESY correlations for 7.

The metabolite lochmolin G (**7**) was also obtained as a colorless oil. Its HRESIMS spectroscopic data (*m/z* 261.1828) suggested the molecular formula C_15_H_26_O_2_, requiring three degrees of unsaturation. IR absorption was observed at 3303 cm^−1^, suggesting the presence of hydroxy group in **7**. In the ^13^C NMR and DEPT spectra (Table 1), signals of four methyls, four sp^3^methylenes, one sp^3^methine, three sp^2^methines, two sp^3^ quaternary carbons, and one sp^2^ quaternary carbons were observed. The ^13^C NMR data of **7** (Table 1) revealed the presence of one trisubstituted and one 1,2-disubstituted carbon-carbon double bond [δc 131.9 (C) and 129.5 (CH); 143.1 (CH) and 128.4 (CH)]. Two hydroxylated carbons (δc 73.0 and 71.9) were also assigned from the ^13^C NMR spectrum. The remaining one degree of unsaturation identified **7** as a cyclic compound. The planar structure of metabolite **7** was elucidated by analysis of ^1^H-^1^H COSY and HMBC correlations (Figure 1). Key HMBC correlations from H_3_-12 to C-7, C-11, and C-13; H_3_-13 to C-7, C-11, and C-12; H_3_-14 to C-1, C-9, and C-10; H_3_-15 to C-3, C-4, and C-5 permitted the establishment of the germacrane skeleton. In the NOESY spectrum of **7** (Figure 4), observation of the NOE correlations between H-6 and H_3_-12, H_3_-13 and H_3_-15, and between H-5 and H-7, suggested that H_3_-15 is α-oriented, and H-7 is β-oriented. The *E* geometries were assigned for the 5,6- and 9,10- double bonds on the basis of the upfield chemical shift of C-14 (δ 16.6) and the large coupling constant between H-5 and H-6 (*J* = 16.0 Hz). Therefore, the relative structure of **7** was established.

**Table 3:** ^1^H NMR spectral data for compounds **5**–**7**.

	5	6	7
1			2.24 m
			2.29 m
2	2.19 m	5.54 dd (3.0, 1.5) *^a^*	1.61 m
	2.24 m		
3	1.78 m	2.25 dd (16.5, 3.0)	1.65 m
	1.98 m	2.55 d (16.5)	1.70 m
5	1.75 d (12.0)	1.96 d (10.0)	5.44 d (16.0)
6	0.50 dd (12.0, 9.5)	0.29 dd (10.0, 9.5)	5.13 dd (16.0, 10.0)
7	0.81 m	0.57 m	2.23 m
8	0.99 m	0.98 m	2.07 m
	2.08 m	1.90 m	2.27 m
9	2.32 dd (13.0, 6.5)	1.59 m	4.90 brd (11.5)
	2.44 t (13.0)	1.89 m	
12	1.07 s	1.03 s	1.11 s
13	1.01 s	1.08 s	1.16 s
14	4.97 s	1.32 s	1.53 s
	4.99 s		
15	1.33 s	1.37 s	1.37 s
1-OOH	7.06 s		

*^a^* *J* values (Hz) in parentheses.

Cytotoxicity of compounds **1**–**7** against the proliferation of a limited panel of cancer cell lines, including human cervical epitheloid (HeLa), liver (SK-Hep1), and melanin (B-16) carcinoma cells, was evaluated. The results showed all of compounds were not cytotoxic toward these three cancer cell lines. The anti-inflammatory activities of **1**–**7** against the accumulation of pro-inflammatory iNOS and COX-2 proteins in RAW264.7 macrophage cells were evaluated by Western blot analysis. It was found that **1**–**7** could not reduce the accumulation of iNOS protein induced by LPS. At a concentration of 1 μM, only compound **1** could reduce the level of LPS-induced COX-2 to 36.6 ± 3.8%. At a concentration of 10 μM, compounds **1**, **3**, and **4** reduced the accumulation of LPS-induced COX-2 to 8.7 ± 4.5%, 61.0 ± 6.0%, and 83.4 ± 6.4%, respectively. At a concentration of 100 μM, **1**–**4** could further reduce the levels of induced COX-2 to 1.7 ± 1.3%, 17.6 ± 2.2%, 32.8 ± 3.2%, and 71.3 ± 7.2%, respectively, in comparison with those of control cells stimulated with LPS only. Thus, compound **1** might be considered to be a promising COX-2 inhibiting agent.

## 3. Experimental Section

### 3.1. General Experimental Procedures

Optical rotations were measured on a JASCO P-1020 polarimeter. IR spectra were recorded on a JASCO FT/IR-4100 infrared spectrophotometer. The NMR spectra were recorded on a Varian Unity INOVA 500 FT-NMR at 500 MHz for ^1^H and 125 MHz for ^13^C, in CDCl_3_ using TMS as internal standard. LRMS and HRMS were obtained by ESI on a Bruker APEX ΙΙ mass spectrometer, or by EI on a JEOL-SX/SX 102A mass spectrometer. Silica gel 60 (Merck, 230–400 mesh) was used for column chromatography. Precoated silica gel plates (Merck, Kieselgel 60 F_254_, 0.2 mm) were used for analytical TLC. High-performance liquid chromatography was performed on a Hitachi L-6250 HPLC apparatus with a merck Hibar Si-60 column (250 × 21 mm, 7 μm).

### 3.2. Animal Material

*Sinularia lochmodes* was collected by hand using SCUBA off the northeast corner of Taiwan, in May 2004, at a depth of 10 to 15 m, and stored in a freezer until extraction. A voucher sample was deposited at the Department of Marine Biotechnology and Resources, National Sun Yat-sen University (specimen No. 20040516-3).

### 3.3. Extraction and Separation

The frozen bodies of *S. lochmodes* (139.4 g) were minced and extracted with ethyl acetate. The organic extract (4.87 g) of the organsim was fractionated by silica gel column chromatography to afford 22 fractions (Fractions A to V). Fraction D, eluted with *n*-hexane–EtOAc (60:1), was purified by normal-phase HPLC (*n*-hexane–EtOAc, 60:1) to afford **1** (3.5 mg). Fraction G, eluted with *n*-hexane–EtOAc (20:1), was purified by normal-phase HPLC (*n*-hexane–EtOAc, 30:1) to afford **2** (1.5 mg). Fraction K, eluted with *n*-hexane–EtOAc (2:1), was purified by normal-phase HPLC (*n*-hexane–EtOAc, 3:1) to afford **3** (2.4 mg), **4** (1.2 mg), and **5** (2.0 mg). Fraction M, eluted with *n*-hexane–EtOAc (1:2), was purified by normal-phase HPLC (*n*-hexane–EtOAc, 1:1) to afford **6** (1.6 mg), and **7** (4.2 mg).

Lochmolin A (**1**): colorless oil; [α]^26^_D_ = −89 (*c* 0.5, CHCl_3_); ^1^H and ^13^C NMR data, see Table 1 and Table 2; EIMS (70 eV) *m*/*z* 234 [M]^+^; HREIMS *m*/*z* 234.1620 (calcd for C_15_H_22_O_2_, 234.1621).

Lochmolin B (**2**): colorless oil; [α]^26^_D_ = −173 (*c* 0.8, CHCl_3_); IR (neat, CHCl_3_) ν_max_ 3437 (broad) cm^−1^; ^1^H and ^13^C NMR data, see Table 1 and Table 2; ESIMS *m*/*z* 289 [M + Na]^+^; HRESIMS *m*/*z* 289.2147 (calcd for C_17_H_30_O_2_Na, 289.2143).

Lochmolin C (**3**): colorless oil; [α]^26^_D_ = −261 (*c* 0.6, CHCl_3_); IR (neat, CHCl_3_) ν_max_ 3437 (broad) cm^−1^; ^1^H and ^13^C NMR data, see Table 1 and Table 2; ESIMS *m*/*z* 275 [M + Na]^+^; HRESIMS *m*/*z* 275.1622 (calcd for C_15_H_24_O_3_Na, 275.1623).

Lochmolin D (**4**): colorless oil; [α]^26^_D_ = −66 (*c* 2.0, CHCl_3_); IR (neat, CHCl_3_) ν_max_ 3406 (broad) cm^−1^; ^1^H and ^13^C NMR data, see Table 1 and Table 2; ESIMS *m*/*z* 275 [M + Na]^+^; HRESIMS *m*/*z* 275.1622 (calcd for C_15_H_24_O_3_Na, 275.1623).

Lochmolin E (**5**): colorless oil; [α]^26^_D_ = −66 (*c* 2.0, CHCl_3_); IR (neat, CHCl_3_) ν_max_ 3448 (broad) cm^−1^; ^1^H and ^13^C NMR data, see Table 1 and Table 3; ESIMS *m*/*z* 275 [M + Na]^+^; HRESIMS *m*/*z* 275.1623 (calcd for C_15_H_24_O_3_Na, 275.1621).

Lochmolin F (**6**): colorless oil; [α]^26^_D_ = −75 (*c* 0.7, CHCl_3_); IR (neat, CHCl_3_) ν_max_ 3396 (broad) cm^−1^; ^1^H and ^13^C NMR data, see Table 1 and Table 3; EIMS (70eV) *m*/*z* 236 [M]^+^; HREIMS *m*/*z* 236.1774 (calcd for C_15_H_24_O_2_, 236.1777).

Lochmolin G (**7**): colorless oil; [α]^26^_D_ = −42 (*c* 0.55, CHCl_3_); IR (neat, CHCl_3_) ν_max_ 3303 (broad) cm^−1^; ^1^H and ^13^C NMR data, see Table 1 and Table 3; ESIMS *m*/*z* 261 [M + Na]^+^; HRESIMS *m*/*z* 261.1828 (calcd for C_15_H_26_O_2_Na, 261.1830).

### 3.4. Cytotoxicity Testing

Cell lines were purchased from the American Type Culture Collection (ATCC). Cytotoxicity assays of compounds **1**–**7** were performed using the Alamar Blue assay [9,10].

### 3.5. **In Vitro** Anti-Inflammatory Assay

Murine RAW264.7 macrophages were purchased from the American Type Culture Collection. The anti-inflammatory assay was modified from known procedure [11,12,13].

## 4. Conclusions

Our present investigation again demonstrated that the Formosan soft coral *Sinularia lochmodes* is a good source of bioactive substances. In our investigation of new and bioactive metabolites from the Formosan soft corals, this is the first study of *S. lochmodes* collected from the northeast corner of Taiwan. The aromadendrane-type compounds **1**–**4**, in particular **1**, might become a promising COX-2 inhibiting agent.
